# Clinical Predictors of Successful Pregnancy After In Vitro Fertilization (IVF): A Comprehensive Systematic Review of Evidence

**DOI:** 10.7759/cureus.100734

**Published:** 2026-01-04

**Authors:** Egbal Lutfi Mohamed Salih, Abduraheem Farah, Hania Mohammed Saeed Mohammed, Hind Suliman Badre Adam, Reem Babkir Altayeb Abdullah

**Affiliations:** 1 Obstetrics and Gynecology, Sabt Alaya Hospital, Alkhaldiyah, SAU; 2 Anatomical Sciences, St. George's University, St. George, GRD; 3 Obstetrics and Gynecology, International University of Africa, Khartoum, SDN; 4 Obstetrics and Gynaecology, Bidyia Hospital, Bidiya, OMN; 5 Obstetrics and Gynecology, Ministry of National Gaurd Health Affairs, Dammam, SAU

**Keywords:** clinical predictors, in vitro fertilization, live birth rate, machine learning, ovarian reserve, pregnancy outcome, systematic review

## Abstract

In vitro fertilization (IVF) success is influenced by a complex interplay of patient- and treatment-related factors. Identifying reliable clinical predictors is crucial for patient counseling and individualized treatment planning. This systematic review aimed to synthesize the most recent evidence on clinical predictors of successful pregnancy after IVF. A comprehensive search of five electronic databases (PubMed, Scopus, Excerpta Medica database (Embase), Web of Science, and ClinicalTrials.gov) was conducted for studies published between 2020 and 2025. Following Preferred Reporting Items for Systematic Reviews and Meta-Analyses (PRISMA) guidelines, studies evaluating clinical predictors of clinical pregnancy or live birth in women undergoing IVF were included. Study selection, data extraction, and risk of bias assessment (using the Quality Assessment of Diagnostic Accuracy Studies-2 (QUADAS-2) tool) were performed independently by two reviewers. A narrative synthesis was conducted due to significant methodological heterogeneity. Eight retrospective observational studies, comprising data from 40,490 IVF/intracytoplasmic sperm injection (ICSI) cycles, were included. Female age was the most consistent and powerful predictor, with a non-linear negative impact, particularly beyond 40 years. Ovarian reserve markers, anti-Müllerian hormone (AMH) and antral follicle count (AFC), were significant, with evidence suggesting AMH better predicts oocyte yield while AFC may better forecast embryo availability. Embryo quality parameters (number of high-quality embryos) were strongly associated with success. Male factors, including total progressive motile sperm count (TPMC) and sperm DNA fragmentation index (DFI), added incremental predictive value. Several studies developed predictive models using both traditional logistic regression and machine learning (ML) algorithms (e.g., eXtreme Gradient Boosting (XGBoost) and Random Forest), which demonstrated high accuracy but raised concerns regarding interpretability and temporal validity. Successful IVF pregnancy is multifactorial, with female age, ovarian reserve, embryo quality, and sperm DNA integrity being key prognostic determinants. While ML-based models show promise, their clinical integration requires rigorous external validation and transparency. Future research should prioritize prospective, multi-center designs and the integration of novel dynamic parameters to advance toward truly personalized prognostic tools in reproductive medicine.

## Introduction and background

Infertility is a significant global health concern, affecting approximately 10%-15% of couples of reproductive age worldwide [1]. Assisted reproductive technologies (ART), a group of medical procedures used to achieve pregnancy, have transformed the management of infertility, with in vitro fertilization (IVF) being the most widely used approach [2]. IVF involves the controlled stimulation of the ovaries, retrieval of oocytes, fertilization outside the body, and subsequent transfer of embryos into the uterus. Since its introduction in 1978, IVF has undergone substantial refinement, with advances in ovarian stimulation protocols, embryo culture systems, and embryo transfer techniques contributing to improved clinical outcomes [3]. Nevertheless, IVF success rates remain variable, and many individuals and couples require multiple treatment cycles without achieving a live birth, emphasizing the clinical importance of identifying factors associated with treatment success.

Successful pregnancy following IVF is determined by a complex interaction of biological, clinical, and procedural factors [4]. Among the most consistently reported predictors are maternal age and indicators of ovarian reserve, such as anti-Müllerian hormone (AMH), a serum marker reflecting the remaining follicular pool, and antral follicle count (AFC), an ultrasound-based measure of recruitable follicles [5]. Embryo quality, commonly assessed using morphological or developmental criteria, and endometrial receptivity, which refers to the uterus’s capacity to allow embryo implantation, also play critical roles in IVF outcomes. In addition, paternal factors, including sperm quality, as well as maternal lifestyle characteristics and comorbid conditions such as obesity, polycystic ovary syndrome (PCOS), and endometriosis, may further influence the probability of conception [6]. Identifying reliable clinical predictors is therefore essential for individualizing treatment strategies, counseling patients regarding prognosis, and optimizing decision-making throughout the IVF process [7].

Over the past decade, a growing body of research has examined predictors of IVF success, encompassing traditional demographic and clinical variables as well as emerging biomarkers and genetic factors [8,9]. However, substantial heterogeneity exists among studies with respect to patient characteristics, IVF protocols, outcome definitions (such as biochemical pregnancy, clinical pregnancy, or live birth), and methodological rigor. This variability limits the comparability of findings and challenges the translation of evidence into routine clinical practice, highlighting the need for a systematic and critically appraised synthesis of the literature.

A comprehensive understanding of clinical predictors of IVF success can support clinicians in stratifying patients according to their likelihood of achieving pregnancy, facilitating personalized treatment planning, and potentially improving the efficiency and cost-effectiveness of IVF programs. Moreover, synthesizing existing evidence can help identify gaps in knowledge, methodological shortcomings, and areas requiring further investigation, thereby informing future research directions in reproductive medicine.

Accordingly, this systematic review aims to synthesize recent evidence on clinical predictors of successful pregnancy following IVF. By focusing on studies published within the last five years (2020-2025), this review seeks to capture contemporary advances in the field, assess the quality and applicability of the available evidence, and provide insights relevant to clinicians, researchers, and interdisciplinary audiences involved in reproductive health and infertility care.

## Review

Methodology

Protocol and Registration

This systematic review was conducted in accordance with the Preferred Reporting Items for Systematic Reviews and Meta-Analyses (PRISMA) guidelines to ensure methodological transparency and reproducibility [10]. A predefined protocol outlining the objectives, eligibility criteria, and methodological approach was developed prior to the literature search. While formal registration with the International Prospective Register of Systematic Reviews (PROSPERO) is ideal, this review was not registered as it was conducted as a rapid synthesis of the most recent evidence (2020-2025) to inform timely clinical guidance. All subsequent review stages were rigorously documented to uphold transparency.

Eligibility Criteria

Eligibility criteria were defined based on the Population, Intervention, Comparison, Outcomes, and Study design (PICOS) framework. Studies were included if they involved adult women undergoing in vitro fertilization (Population), evaluated clinical predictors associated with pregnancy outcomes (Intervention/Exposure), compared groups with successful versus unsuccessful pregnancy outcomes (Comparator), and reported clinical pregnancy or live birth rates as primary outcomes (Outcome). Both prospective and retrospective original studies, including observational cohorts and clinical trials, were considered. Only studies published in English between 2020 and 2025 were included to ensure the incorporation of the most recent and relevant evidence. Review articles, editorials, conference abstracts, and case reports were excluded.

Information Sources and Search Strategy

A comprehensive literature search was performed using multiple electronic databases, including PubMed, Scopus, Excerpta Medica database (Embase), Web of Science, and ClinicalTrials.gov. The search strategy combined keywords and MeSH terms related to “IVF,” “in vitro fertilization,” “clinical predictors,” and “pregnancy outcomes,” with Boolean operators applied to maximize retrieval. The last search was conducted on 15 November 2025. Reference lists of relevant articles were manually screened to identify additional eligible studies. The full search strings for all databases are provided in Appendix A.

Study Selection

All retrieved records were imported into EndNote X21 (Clarivate, London, United Kingdom) to manage references and remove duplicates. Two independent reviewers screened titles and abstracts to identify potentially eligible studies. Full-text articles were subsequently assessed for eligibility based on the predefined inclusion and exclusion criteria. Any discrepancies between reviewers were resolved through discussion or consultation with a third reviewer to ensure unbiased study selection.

Data Collection Process and Data Items

Data extraction was performed independently by two reviewers using a structured data collection form. Extracted data included country, study design, sample size, patient characteristics (such as age and infertility type), clinical predictors evaluated, methods of predictor assessment, and primary pregnancy outcomes. Key findings and statistical measures were also recorded to facilitate qualitative synthesis.

Risk of Bias Assessment

The quality of included studies and risk of bias were assessed using the Quality Assessment of Diagnostic Accuracy Studies-2 (QUADAS-2) tool [11], which evaluates patient selection, index test, reference standard, and flow and timing. Applicability concerns were also considered. Discrepancies in risk of bias assessment were resolved through consensus between reviewers.

Data Synthesis

Due to substantial heterogeneity among the included studies-including variability in patient populations, predictor definitions, study designs, outcome measures, and follow-up durations, a formal meta-analysis was not feasible. Instead, a narrative synthesis was conducted to summarize and compare clinical predictors of successful pregnancy after IVF. Findings were organized to highlight consistent associations and identify gaps in the current evidence base.

Results

Studies Selection Process

**Figure 1:**
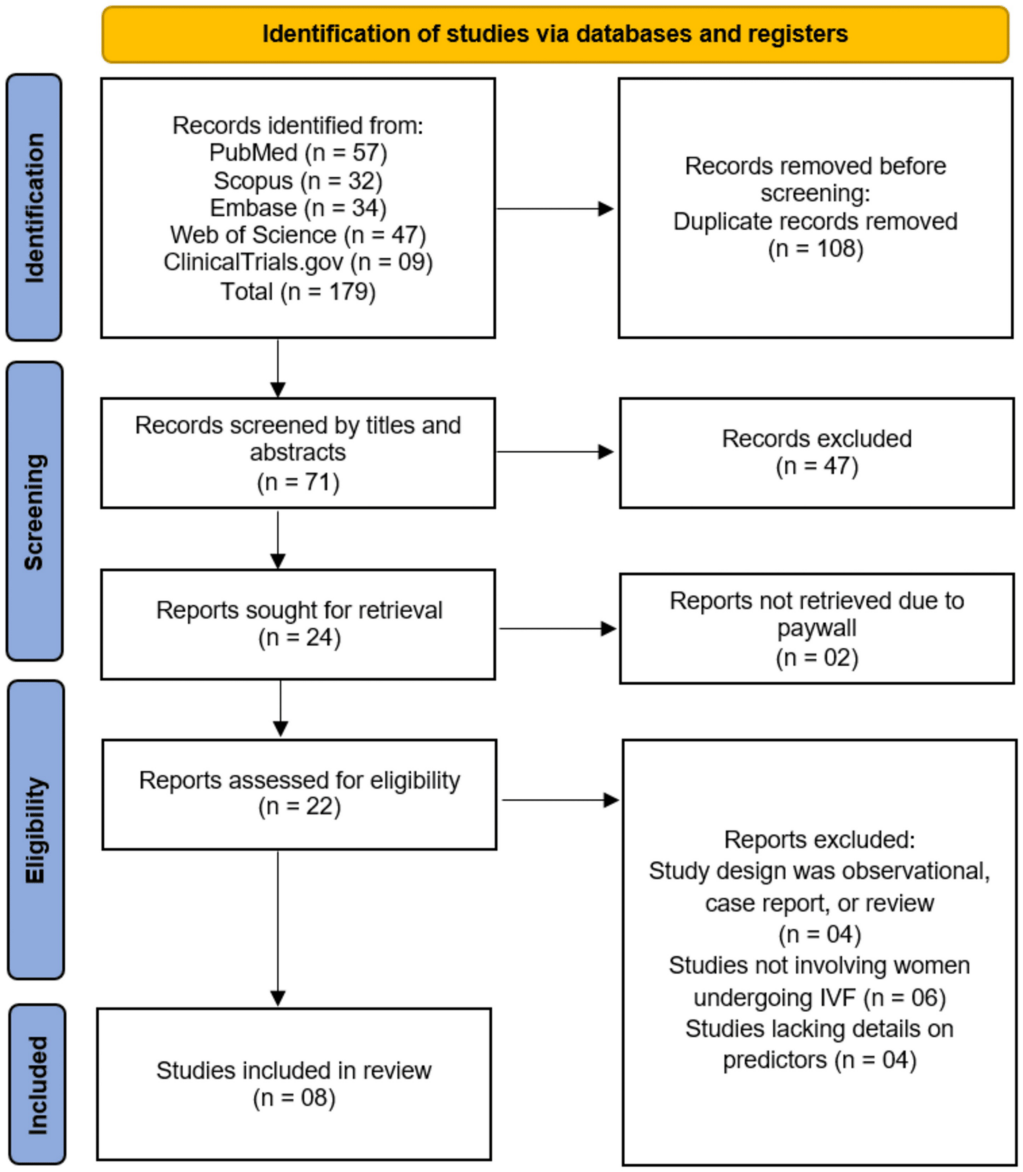
A PRISMA flowchart outlining the study selection process PRISMA: Preferred Reporting Items for Systematic Reviews and Meta-Analyses; Embase: Excerpta Medica database; IVF: In Vitro Fertilization

The systematic search of five electronic databases (PubMed, Scopus, Embase, Web of Science, and ClinicalTrials.gov) initially identified 179 potential records. After the removal of 108 duplicate records, a total of 71 unique citations were screened based on their titles and abstracts. This initial screening led to the exclusion of 47 records that did not meet the broad inclusion criteria. The remaining 24 reports were sought for full-text retrieval, of which two could not be retrieved due to paywall restrictions. Therefore, 22 full-text articles were assessed for eligibility. Upon detailed evaluation, a further 14 articles were excluded for the following reasons: the study design was observational, a case report, or a review without original predictive data (n=4); the study did not involve women undergoing IVF (n=6); or the study lacked sufficient details on the clinical predictors of interest (n=4). Consequently, eight studies met all pre-defined eligibility criteria and were included in the final systematic review [12-19]. The selection process is summarized in the PRISMA flow diagram (Figure 1).

Study Characteristics and Populations

A total of eight studies [12-19] published between 2022 and 2025 were included in this systematic review, providing data from 40,490 IVF or intracytoplasmic sperm injection (ICSI) cycles. The characteristics of these studies are summarised in Table 1. All studies employed a retrospective observational design, with sample sizes ranging from 369 to 32,306 cycles. The primary outcomes assessed across the studies included clinical pregnancy, live birth, cumulative live birth (CLB), and fertilization failure. The predictors evaluated encompassed a wide range of female, male, and treatment-cycle factors, with analytical methods spanning traditional logistic regression to advanced machine learning (ML) techniques.

**Table 1:** Summary of Included Studies Evaluating Clinical Predictors of Pregnancy Outcomes Following IVF IVF-ET: In Vitro Fertilization and Embryo Transfer; ICSI: Intracytoplasmic Sperm Injection; CLB: Cumulative Live Birth; TPMC: Total Progressive Motile Count; DFI: DNA Fragmentation Index; DOR: Diminished Ovarian Reserve; AMH: Anti-Müllerian Hormone; AFC: Antral Follicle Count; FSH: Follicle-Stimulating Hormone; LH: Luteinizing Hormone; OSI: Ovarian Sensitivity Index; COS: Controlled Ovarian Stimulation; hCG: Human Chorionic Gonadotropin; LBO: Live Birth Outcome; ANN: Artificial Neural Network; AVM: Adaptive Vector Machine; HCT: Hematocrit; P: Progesterone; D3: Day 3 embryos.

Study (Author, Year)	Country	Study Design	Sample Size (n)	Patient Population (e.g., Age, Infertility Type)	Predictors Evaluated	Predictor Measurement Method	Primary Outcome	Key Findings
Bai et al. (2025) [12]	China (Inner Mongolia)	Retrospective observational study with predictive model development	2,625	Women undergoing fresh IVF-ET cycles (2016–2022)	Multiple clinical features related to IVF-ET treatment	Clinical data preprocessing and machine-learning–based feature analysis using XGBoost and LightGBM models	Clinical pregnancy and live birth	XGBoost achieved the highest accuracy for predicting clinical pregnancy; LightGBM best predicted live birth
Zhu et al. (2025) [13]	China	Retrospective cohort study	1457	Women with endometriosis undergoing IVF/ICSI	Age; concurrent adenomyosis; duration of infertility; number of fertilized oocytes; number of high-quality embryos	Clinical record review; univariate analysis and multivariable logistic regression	CLB rate	Concurrent adenomyosis was associated with reduced CLB; longer infertility duration decreased CLB; higher numbers of fertilized oocytes and high-quality embryos increased CLB; the predictive nomogram showed good discrimination
Wang et al. (2025) [14]	China	Retrospective single-center observational study	691 IVF cycles (594 c-IVF, 97 rescue ICSI)	Infertile couples undergoing conventional IVF; variables included female age, female BMI, male age, semen quality parameters, and infertility duration	Female age, female BMI, male age, sperm concentration, TPMC, DFI, and infertility duration	Clinical records and laboratory semen analysis; machine learning–based modeling	Fertilization failure in conventional IVF cycles	Logistic regression showed the best predictive performance; protective predictors were male age and TPMC, while female BMI and sperm DNA fragmentation index were significant risk factors for fertilization failure
Zeng et al. (2025) [15]	China	Retrospective observational study	1,403 IVF/ICSI cycles (1,039 patients); 441 fresh embryo transfer cycles	Patients with DOR; subgroup analyses by age (<40 vs ≥40 years)	AMH, AFC, basal FSH, age, D3 available cleavage-stage embryos, D3 top-quality embryos	Serum hormone assays (AMH, basal FSH), transvaginal ultrasound (AFC), embryo morphological grading on Day 3, logistic regression with ROC analysis	Oocyte retrieval, D3 available cleavage-stage embryos, clinical pregnancy (fresh cycle), viable blastocyst formation	AMH was a stronger predictor of oocyte retrieval than AFC and basal FSH; AFC showed superior predictive accuracy for obtaining D3 available embryos; D3 top-quality embryos predicted clinical pregnancy better than age in patients <40 years, whereas age was more predictive in patients ≥40 years
Bereczki et al. (2025) [16]	Hungary	Retrospective cohort study with model development and validation	1243 IVF/ICSI cycles (development cohort); 92 cycles	Women and couples undergoing IVF/ICSI; baseline characteristics collected at first consultation, including female age, BMI, hormonal profile, infertility duration, and male semen parameters	Female age, AMH, BMI, FSH, LH, infertility duration, sperm concentration, sperm motility, and other baseline clinical variables	Routine preprocedural clinical and laboratory measurements were collected at the first IVF consultation	IVF success per cycle	A nine-variable model showed strong predictive performance, with consistent discrimination in external validation. Female age was the strongest predictor, while AMH and BMI were key contributors; male factors added incremental predictive value.
Xia et al. (2024) [17]	China	Retrospective cohort study	32,306 complete IVF cycles (29,023 couples)	Women/couples undergoing IVF (2014–2020); varying female age; primary and secondary infertility; tubal factor, male factor, scarred uterus; multiple prior IVF attempts	Female age, AFC, BMI, number of previous IVF attempts, previous embryo transfer failures, infertility type, tubal factor, male factor, scarred uterus, number of oocytes retrieved	Clinical and laboratory measurements during pre-treatment, post-stimulation, and post-treatment phases; non-linear effects modeled using restricted cubic splines	CLBR	Three stage-specific predictive models showed good discrimination. Female age and oocyte number had non-linear associations with CLBR. Internal validation showed good calibration; temporal validation showed reduced accuracy, possibly due to improvements in IVF practices over time.
Liu et al. (2024) [18]	China	Retrospective cohort	1405	Patients undergoing IVF; age not specified, infertility type not specified	Age, OSI, COS regimen, Gn starting dose, endometrial thickness on HCG day, Progesterone value on HCG day, embryo transfer strategy	Clinical records; IVF cycle data; laboratory measurements	LBO	Seven predictors significantly associated with LBO; ANN and SVM models built, SVM performed best; models can predict LBO and assist in customizing embryo transfer strategy
Yang et al., (2022) [19]	China	Nested Case-Control	369	Women receiving IVF-ET (age and infertility type not specified)	Age, BMI, Number of cycles, HCT, LH, P, Endometrial thickness, FSH	Clinical/laboratory measurement of hormones, hematocrit, BMI, endometrial thickness; IVF treatment records	Clinical pregnancy after IVF-ET	Age, BMI, P, FSH were positively associated with clinical pregnancy; three cycles, HCT, LH, and endometrial thickness were negatively associated; the random forest model validated to predict clinical pregnancy outcomes.

The included studies were predominantly conducted in China (n=7), with one study from Hungary [16]. Patient populations varied, including general IVF populations [12, 17, 18], women with specific conditions such as endometriosis [13] or diminished ovarian reserve (DOR) [15], and couples undergoing conventional IVF [14]. Study designs were exclusively retrospective, comprising cohort studies [13, 16-18], model development studies [12, 14, 16], and one nested case-control study [19]. The large variation in sample size is notable, with Xia et al. [17] analyzing a substantial cohort of over 32,000 cycles, providing considerable statistical power.

Predictors of Pregnancy and Live Birth Outcomes

Female age was consistently identified as a critical predictor across multiple studies. It was reported as the strongest individual predictor of IVF success in several analyses [16, 17]. The relationship was often non-linear, with advanced maternal age (typically ≥40 years) being associated with significantly reduced probabilities of success [15, 17]. Ovarian reserve markers, including AMH and AFC, were also significant predictors. Zeng et al. [15] found AMH to be a stronger predictor of oocyte retrieval than AFC and basal FSH in women with DOR, while AFC was superior for predicting the availability of Day-3 embryos. Bereczki et al. [16] confirmed AMH as a key contributor in predictive models.

Other female factors played important roles. BMI was identified as a significant risk factor, with higher BMI associated with reduced success in some studies [14, 16, 19]. For patients with endometriosis, Zhu et al. [13] found that concurrent adenomyosis was independently associated with a reduced CLB rate. Endometrial thickness was another recurring factor, though its association was inconsistent; Yang et al. [19] reported a negative association with clinical pregnancy, whereas Liu et al. [18] included it in their predictive model for live birth.

Male factors contributed incremental predictive value. Wang et al. [14] identified total progressive motile sperm count (TPMC) as a protective factor against fertilization failure, while sperm DNA fragmentation index (DFI) was a significant risk factor. Bereczki et al. [16] also noted that incorporating male semen parameters improved model performance.

Embryological parameters were strongly predictive. The number of fertilized oocytes and the number of high-quality or top-quality embryos were positively associated with CLB and clinical pregnancy rates [13, 15]. Zeng et al. [15] highlighted that in patients under 40 years, the quality of Day-3 embryos was a better predictor of clinical pregnancy than age itself.

Performance and Validation of Predictive Models

Several studies developed and tested multivariable predictive models. Traditional statistical methods like logistic regression were used effectively [13-15]. For instance, Zhu et al. [13] developed a nomogram based on logistic regression that showed good discrimination for predicting CLB in endometriosis patients.

A prominent trend was the application of ML algorithms, which generally demonstrated high predictive accuracy. Bai et al. [12] compared multiple ML techniques, reporting that the XGBoost model achieved the highest accuracy for predicting clinical pregnancy, while LightGBM was best for predicting live birth. Similarly, Liu et al. [18] found that a support vector machine (SVM) model outperformed an artificial neural network (ANN) for predicting live birth outcomes. Yang et al. [19] validated a random forest model for predicting clinical pregnancy. Bereczki et al. [16] developed a nine-variable model, likely using ML methods, which showed strong and consistent predictive performance upon external validation.

However, model performance over time can be variable. Xia et al. [17] developed stage-specific predictive models with good initial discrimination and calibration but noted that temporal validation showed reduced accuracy, potentially due to evolving IVF laboratory and clinical practices.

The evidence synthesized from these eight studies indicates that successful pregnancy outcomes following IVF are multifactorial. The most robust predictors include female age, ovarian reserve markers (AMH, AFC), embryo quality parameters, and specific male factor semen analyses. Predictive modelling using both traditional regression and advanced ML techniques shows promise for individualized prognosis, though the generalizability and temporal stability of these models require further validation.

Risk of Bias Assessment

The methodological quality of the eight included studies was assessed using the QUADAS-2 tool, adapted for predictive model studies. Regarding risk of bias, most studies demonstrated low risk in the domains of the index test (predictors and model development), reference standard (pregnancy outcomes), and flow and timing [13, 15-17]. However, concerns were identified in patient selection for three studies due to either unclear inclusion criteria [12] or insufficient detail on key population characteristics such as age and infertility type [18, 19], resulting in a high risk of selection bias. Furthermore, one study was rated high risk in the flow and timing domain because it analyzed IVF cycles rather than independent patients, potentially violating statistical independence [14]. In terms of applicability to the review question, all studies were deemed to have low concern regarding the predictors and outcomes evaluated. Nonetheless, applicability regarding patient selection was of great concern for two studies [18, 19] due to their poorly described populations, limiting generalizability. Overall, while the core predictive methodologies in most studies [13-17] appear robust, the findings from Bai et al. [12], Liu et al. [18], Yang et al. [19], and Wang et al. [14] should be interpreted with caution due to the identified biases (Table 2).

**Table 2:** Risk of Bias Assessment for the Included Studies Using the Quality Assessment of Diagnostic Accuracy Studies-2 (QUADAS-2) Tool

Study (Author, Year)	Risk of Bias				Applicability Concerns		
	Patient Selection	Index Test	Reference Standard	Flow & Timing	Patient Selection	Index Test	Reference Standard
Bai et al. (2025) [12]	⚫ High	⚪ Low	⚪ Low	⚪ Low	⚪ Low	⚪ Low	⚪ Low
Zhu et al. (2025) [13]	⚪ Low	⚪ Low	⚪ Low	⚪ Low	⚪ Low	⚪ Low	⚪ Low
Wang et al. (2025) [14]	⚪ Low	⚪ Low	⚪ Low	⚫ High	⚪ Low	⚪ Low	⚪ Low
Zeng et al. (2025) [15]	⚪ Low	⚪ Low	⚪ Low	⚪ Low	⚪ Low	⚪ Low	⚪ Low
Bereczki et al. (2025) [16]	⚪ Low	⚪ Low	⚪ Low	⚪ Low	⚪ Low	⚪ Low	⚪ Low
Xia et al. (2024) [17]	⚪ Low	⚪ Low	⚪ Low	⚪ Low	⚪ Low	⚪ Low	⚪ Low
Liu et al. (2024) [18]	⚫ High	⚪ Low	⚪ Low	⚪ Low	⚫ High	⚪ Low	⚪ Low
Yang et al. (2022) [19]	⚫ High	⚪ Low	⚪ Low	⚪ Low	⚫ High	⚪ Low	⚪ Low

Discussion

This systematic review synthesises evidence from eight recent studies exploring the clinical predictors of successful pregnancy following IVF. The findings collectively underscore the multifactorial nature of IVF success, identifying a core set of robust prognostic factors while highlighting the burgeoning role of ML in crafting personalised predictive models. The most consistent and potent predictors emerging from this synthesis are female age, markers of ovarian reserve (AMH and AFC), embryo quality parameters, and specific semen analysis metrics. These factors align with the fundamental biological pillars of reproduction: ovarian capacity, oocyte and embryo competence, and spermatozoal contribution. The pronounced and often non-linear negative impact of advanced female age, particularly beyond 40 years, is a recurrent theme, corroborated by multiple studies in this review [15-17]. This finding is biologically intuitive, reflecting the well-documented decline in oocyte quantity and quality, increased aneuploidy rates, and altered endometrial receptivity associated with aging. Our review reinforces that age is not merely a demographic variable but the single strongest clinical predictor, a conclusion that echoes decades of epidemiological and clinical research in reproductive medicine.

The assessment of ovarian reserve through AMH and AFC has become a cornerstone of pre-IVF evaluation, and our findings provide nuanced insights into their predictive utility. Zeng et al. [15] offered a valuable distinction, demonstrating that in patients with DOR, AMH was superior for predicting oocyte yield, while AFC was more accurate for forecasting the availability of viable Day-3 embryos. This suggests these markers, while correlated, may inform different stages of the IVF process, with AMH reflecting the follicular pool and AFC offering a more direct, cycle-specific snapshot. This is consistent with existing literature that positions AMH as a robust marker of ovarian aging and quantitative response, while AFC may have additional value in predicting oocyte maturity. The confirmation by Bereczki et al. [16] that AMH is a key contributor in a multi-factorial model further solidifies its clinical relevance. However, it is crucial to contextualize these markers; they are excellent predictors of ovarian response but are imperfect surrogates for oocyte quality and ultimate live birth, a distinction highlighted by the independent predictive value of embryo quality parameters in our reviewed studies [13, 15].

The significance of embryo morphology, specifically the number of high-quality or top-quality embryos, as a powerful predictor of CLB and clinical pregnancy [13, 15] underscores a critical transition in the prognostic timeline: from pre-treatment potential to actual laboratory outcome. Zeng et al.'s [15] finding that in younger patients (<40 years), day-3 embryo quality surpassed female age as a predictor of clinical pregnancy is particularly instructive. It implies that once a cohort of good-quality embryos is obtained from a young ovary, the age-related decline in endometrial receptivity may be a lesser barrier to success than the inherent viability of the embryos themselves. This aligns with the broader paradigm shift in IVF towards embryo-centric selection, championed by the widespread adoption of comprehensive chromosome screening (PGS/PGT-A), which seeks to directly assess embryonic competence beyond morphology. Our reviewed studies, focused on clinical and embryological predictors, complement the genetic landscape, suggesting that even without genetic testing, traditional embryo grading retains significant prognostic power.

The inclusion of male factors in several studies [14, 16] marks an important evolution from a predominantly female-centric prediction model to a more holistic couple-focused approach. Wang et al. [14] identified TPMC as protective and sperm DFI as a significant risk factor for fertilization failure in conventional IVF cycles. This reinforces a growing body of evidence that sperm contribution extends beyond simple motility and morphology to include genomic integrity. Bereczki et al. [16] further noted that male parameters added incremental predictive value, challenging the historical underestimation of the male role in IVF outcomes beyond severe male factor infertility. These findings are supported by external literature, such as the work of Osman et al. [20], which demonstrated that high sperm DNA fragmentation negatively impacts blastocyst development and pregnancy rates, and the systematic review by Cissen et al. [21], which confirmed the clinical value of advanced sperm function tests. Our review consolidates the view that comprehensive male assessment, including DFI, should be integrated into pre-IVF prognostic evaluations.

A striking trend across the included studies is the comparative application of ML algorithms alongside traditional logistic regression. Studies by Bai et al. [12], Liu et al. [18], and Yang et al. [19] demonstrated that models like XGBoost, SVM, and Random Forest could achieve high predictive accuracy, often outperforming or complementing conventional statistics. This aligns with a broader movement in medicine towards leveraging complex, non-linear algorithms to handle high-dimensional clinical data. For instance, the work of Salih et al. [22] in Fertility and Sterility successfully used deep learning to predict live birth from time-lapse embryo imaging data, surpassing embryologist assessment. Similarly, a study by Liu et al. [23] developed an ML model integrating clinical and genetic data to predict IVF success with high discrimination. The advantage of ML lies in its ability to model intricate interactions and non-linear relationships without pre-specified assumptions, as seen in Xia et al.'s [17] use of restricted cubic splines to capture the non-linear effect of age and oocyte number. However, the "black box" nature of some ML models can limit clinical interpretability compared to the transparent odds ratios of a logistic regression nomogram, as produced by Zhu et al. [13]. The critical challenge, noted by Xia et al. [17], is model decay over time due to evolving clinical practices, underscoring the need for continuous external validation and updating, a requirement even more pressing for complex ML models to ensure their generalizability beyond the development cohort.

When contextualized within the wider existing literature, our synthesized findings both confirm and refine established knowledge. The primacy of female age is universally acknowledged, mirroring conclusions from large registry analyses such as those of the Society for Assisted Reproductive Technology (SART) database in the United States and the Human Fertilisation and Embryology Authority (HFEA) data in the United Kingdom. Our observation on the differential predictive strengths of AMH and AFC finds resonance in the meta-analysis by Tal & Seifer [24], which also debated their respective roles in predicting poor response versus live birth. The strong predictive value of embryo quality is a cornerstone of IVF practice, supported by landmark studies such as the one by Qiu et al. [25], which established the correlation between blastocyst morphology and implantation potential. Furthermore, the incremental value of male factors corroborates the conclusions of the systematic review by Goyal et al. [26] on sperm DFI and IVF outcomes. The promising results from ML applications in our review are part of a rapidly expanding field, evidenced by the pioneering work of Zhang et al. [27], who developed an AI platform to predict implantation potential from static embryo images. However, our review also highlights gaps often present in the existing literature, such as the under-reporting of CLB rates, addressed by Zhu et al. [13] and Xia et al. [17], which provide a more patient-centered outcome than singleton cycle-based pregnancy rates.

Despite these coherent findings, the risk of bias assessment necessitates a cautious interpretation. The high risk of selection bias in studies by Bai et al. [12], Liu et al. [18], and Yang et al. [19], primarily due to unclear inclusion criteria or poorly described populations, means their reported predictor strengths or model accuracies may not be fully generalizable. Similarly, the analysis of cycles rather than unique patients by Wang et al. [14] introduces a unit-of-analysis error that could inflate the statistical significance of their findings. These methodological limitations are not uncommon in retrospective, single-center model development studies, which form the bulk of the current evidence. They highlight a critical need for future research to adhere to stricter reporting guidelines, such as the Transparent Reporting of a multivariable prediction model for Individual Prognosis Or Diagnosis (TRIPOD) statement for predictive model studies, and to prioritize prospective, multicenter designs with explicit protocols for patient selection and clear documentation of population characteristics. The high applicability concerns for two studies [18, 19] further remind us that predictive models are only as good as the population they were derived from; a model built on an obscure population has limited utility in broader clinical practice.

Limitations

This systematic review has several limitations. First, the exclusive inclusion of retrospective studies inherently carries risks of confounding and bias that cannot be fully mitigated, despite our quality assessment. Second, all but one study were conducted in China, potentially limiting the generalizability of the findings to other ethnic and clinical practice populations, as ovarian reserve markers and treatment protocols can vary globally. The Hungarian study [16] offers a valuable but singular point of external validation. Third, the heterogeneity in predictor selection, outcome definitions (e.g., clinical pregnancy vs. CLB), and modelling techniques precluded a formal meta-analysis, limiting our synthesis to a narrative summary. Fourth, the search was restricted to published literature, introducing the possibility of publication bias, where studies with negative or null findings may be underrepresented. Finally, the rapidly evolving field of IVF, with continuous improvements in laboratory techniques (e.g., vitrification, PGT-A, time-lapse imaging) and stimulation protocols, means that predictors identified from cycles performed several years ago may have changing relevance, as hinted at by the temporal validation results of Xia et al. [17].

## Conclusions

This systematic review affirms that successful pregnancy after IVF is predicated on a constellation of clinical factors, with female age, ovarian reserve markers, embryo quality, and sperm DNA integrity standing out as key prognostic determinants. The evidence demonstrates a promising adjunct role for ML models in synthesizing these variables into personalized predictions, though they have yet to surpass the clinical interpretability and established utility of traditional regression-based tools like nomograms. The path forward requires a concerted effort to strengthen the methodological rigor of predictive research through prospective, multi-center collaborations and adherence to robust reporting standards. Future studies should strive to integrate novel, dynamically assessed parameters, such as time-lapse morphokinetics, proteomic profiles of endometrial fluid, or advanced sperm functional assays, into these predictive frameworks. Ultimately, the goal is to move beyond population-level statistics to provide couples with truly individualized prognostic estimates, thereby guiding clinical decision-making, managing expectations, and optimizing the allocation of resources in the emotionally and financially demanding journey of IVF.

## Appendices

Appendix A 

**Table 3:** Search Strategy for Databases MEDLINE: Medical Literature Analysis and Retrieval System Online; Embase: Excerpta Medica database

Database	Search String
PubMed (MEDLINE)	("In Vitro Fertilization"[Mesh] OR IVF OR "Assisted Reproductive Technology" OR ART) AND ("Pregnancy Outcome"[Mesh] OR pregnancy OR "clinical pregnancy" OR "live birth") AND (predict* OR determinant* OR factor* OR prognostic* OR predictor*)
Scopus	TITLE-ABS-KEY ( "in vitro fertilization" OR IVF OR "assisted reproductive technology" OR ART ) AND TITLE-ABS-KEY ( pregnancy OR "clinical pregnancy" OR "live birth" OR outcome* ) AND TITLE-ABS-KEY ( predict* OR determinant* OR factor* OR prognostic* )
Embase	('in vitro fertilization'/exp OR IVF OR 'assisted reproductive technology') AND ('pregnancy outcome'/exp OR pregnancy OR 'clinical pregnancy' OR 'live birth') AND (predict* OR determinant* OR factor* OR prognostic*)
Web of Science (Core Collection)	TS=("in vitro fertilization" OR IVF OR "assisted reproductive technology" OR ART) AND TS=(pregnancy OR "clinical pregnancy" OR "live birth" OR outcome*) AND TS=(predict* OR determinant* OR factor* OR prognostic*)
ClinicalTrials.gov	Condition or disease: Infertility OR IVF AND Other terms: pregnancy outcome OR live birth OR predictor OR prognostic
